# A guide to selecting high-performing antibodies for MMP7 (UniProt ID: P09237) for use in western blot and immunoprecipitation

**DOI:** 10.12688/f1000research.175973.1

**Published:** 2026-01-19

**Authors:** Michael Biddle, Jemma Cooper, Carolyn Jones, Katie Dixon, Harvinder Virk

**Affiliations:** 1 University of Leicester College of Life Sciences, Leicester, England, UK; 2 Institute for Precision Health, University of Leicester, Leicester, England, UK

**Keywords:** P09237, MMP7, Matrilysin, matrix metallopeptidase 7, antibody validation, western blot, immunoprecipitation, interstitial lung disease, idiopathic pulmonary fibrosis

## Abstract

Matrix metallopeptidase 7 (MMP7, also known as matrilysin) is a secreted zinc-dependent endopeptidase implicated in extracellular matrix remodelling and fibrotic processes. Elevated MMP7 expression is a hallmark of idiopathic pulmonary fibrosis and other interstitial lung diseases, where it has emerged as a candidate biomarker for disease progression. Identifying high-quality research antibodies is therefore essential to enable robust investigation of MMP7 biology and its translational potential. In this study, we systematically evaluated ten commercial antibodies for western blot and immunoprecipitation using a standardized knockout validation approach in human A549 cells, comparing readouts in MMP7 knockout lines with isogenic parental controls. These experiments form part of a larger collaborative initiative to address antibody reproducibility by characterizing commercial antibodies for human proteins and making the results openly available to the community. While antibody use and protocol conditions will vary between laboratories, this report provides a resource to guide selection of the most suitable reagents for studies of MMP7 in health and disease.

## Introduction

Interstitial lung diseases (ILDs) comprise a heterogenous group of pulmonary disorders characterised by varying degrees of inflammation and fibrosis of the lung interstitium, with idiopathic pulmonary fibrosis (IPF) representing one of the most severe and progressive forms. Matrix Metallopeptidase 7 (MMP7, also known as Matrilysin), encoded by the *MMP7* gene, is a secreted zinc-dependent endopeptidase that degrades multiple components of the extracellular matrix. ^1^ In IPF, MMP7 is predominantly expressed by hyperplastic epithelium, with increased protein abundance in lung base tissue compared with non-diseased controls. ^2^ The role of MMP7 is thought to be pro-fibrotic, as *MMP7* knockout mice are protected from bleomycin-induced pulmonary fibrosis. ^3^ Clinically, elevated levels of MMP7 in serum and bronchoalveolar lavage fluid have been associated with disease progression and severity, supporting its potential as a promising biomarker for both diagnosis and prognosis. ^4^

This research is part of a broader collaborative initiative in which academics, funders and commercial antibody manufacturers are working together to address antibody reproducibility issues by characterising commercial antibodies for human proteins using standardized protocols ^5^ and openly sharing the data. ^6^ Here we evaluated the performance of ten commercial antibodies for MMP7 for use in western blot and immunoprecipitation, enabling biochemical and cellular assessment of MMP7 properties and function. The platform for antibody characterisation used to carry out this study was endorsed by a committee of industry academic representatives. It consists of identifying human cell lines with adequate target protein expression and the development/contribution of equivalent knockout (KO) cell lines, followed by antibody characterisation procedures using most commercially available renewable antibodies against the corresponding protein. The standardised consensus antibody characterisation protocols are openly available on Protocols.io (DOI: dx.doi.org/10.21203/rs.3.pex-2607/v1).

The authors do not engage in result analysis or offer explicit antibody recommendations. Our primary aim is to deliver top-tier data to the scientific community, grounded in Open Science principles. This empowers experts to interpret the characterisation data independently, enabling them to make informed choices regarding the most suitable antibodies for their specific experimental needs. Guidelines on how to interpret the antibody characterisation data found in this study are featured on the YCharOS gateway. ^7^

## Results and discussion

Our standard protocol involves comparing readouts from WT (wild type) and KO cells. ^8, 9^ The first step is to identify a cell line(s) that expresses sufficient levels of a given protein to generate a measurable signal using antibodies. To this end, we examined the DepMap transcriptomics database to identify all cell lines that express the target at levels greater than 2.5 log _2_ (transcript per million “TPM” + 1), which we have found to be a suitable cut-off (Cancer Dependency Map Portal, RRID:SCR_017655). The cell line A549 expresses the MMP7 transcript at 4.3 log _2_ (TPM+1) and thus was identified as a suitable cell line and was modified by CRISPR/Cas9 to KO the corresponding *MMP7* gene ( Table 1).

**Table 1.** Summary of the cell lines used.

Institution	Catalogue number	RRID (Cellosaurus)	Cell line	Genotype
Abcam	ab288558	CVCL_0023	A549	WT
University of Leicester	-	CVCL_F0RJ	A549	MMP7 KO

**Figure 1.**
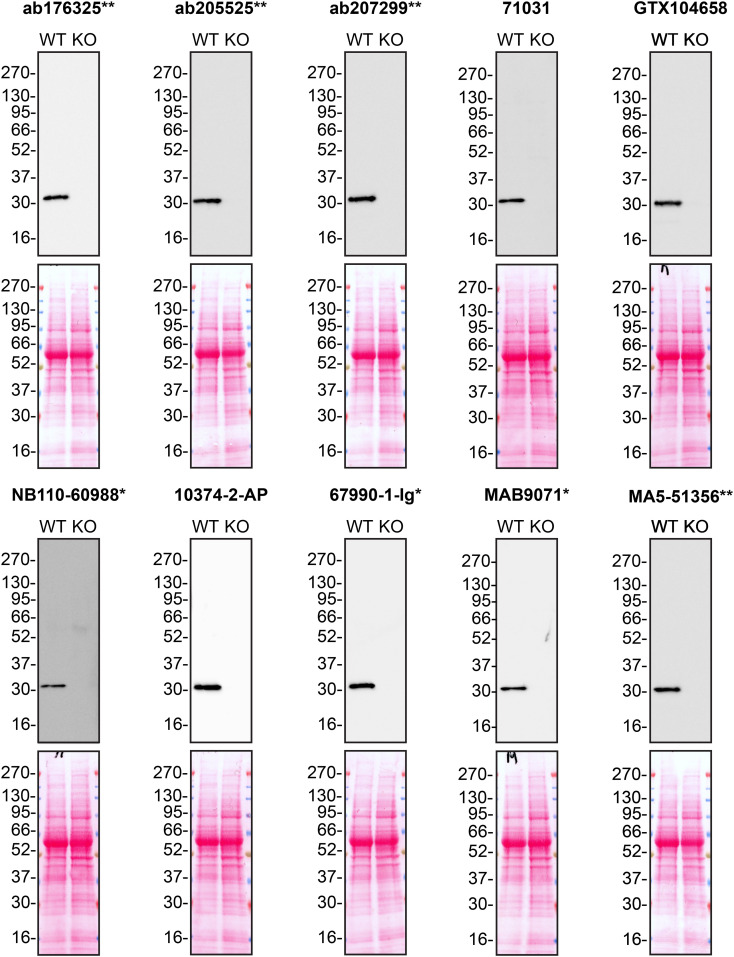
MMP7 antibody screening by western blot using concentrated conditioned culture media. Culture media from A549 WT and *MMP7* KO cells were collected, and 30 μg of protein was processed for western blot with the indicated MMP7 antibodies. The Ponceau stained transfers of each blot are presented to show equal loading of WT and KO samples. Antibody dilutions were chosen according to the recommendations of the antibody supplier. Antibody dilutions used: ab176325** at 1/1000, ab205525** at 1/1000, ab207299** at 1/1000, 71031 at 1/1000, GTX104658 at 1/1000, NB110-60988* at 1/1000, 10374-2-AP at 1/1000, 67990-1-Ig* at 1/2000, MAB9071* at 1/500 and MA5-51356** at 1/1000. Predicted band size: 29.7 kDa. *Monoclonal antibody; **Recombinant antibody.

**Figure 2.**
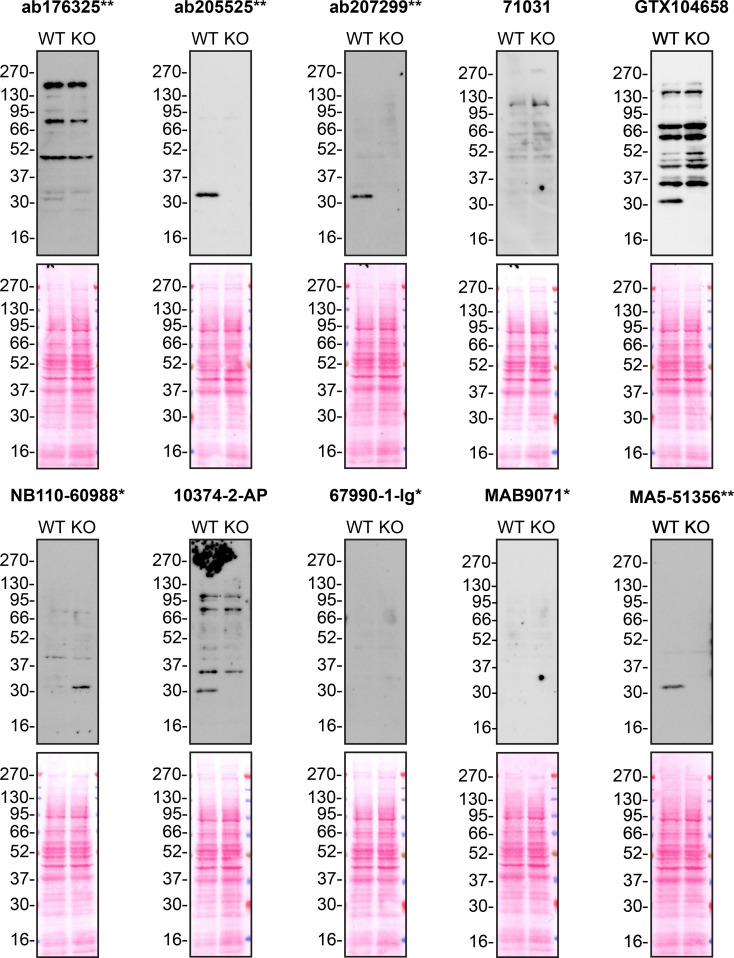
MMP7 antibody screening by western blot using protein lysates. Protein lysates from A549 WT and *MMP7* KO cells were collected, and 30μg of protein was used for western blot with the indicated MMP7 antibodies. The Ponceau stained transfers of each blot are presented to show equal loading of WT and KO samples. Antibody dilutions were chosen according to the recommendations of the antibody supplier. Antibody dilutions used: ab176325** at 1/1000, ab205525** at 1/1000, ab207299** at 1/1000, 71031 at 1/1000, GTX104658 at 1/1000, NB110-60988* at 1/1000, 10374-2-AP at 1/1000, 67990-1-Ig* at 1/2000, MAB9071* at 1/500 and MA5-51356** at 1/1000. Predicted band size: 29.7 kDa. *Monoclonal antibody; **Recombinant antibody.

According to the UniProt database, MMP7 is secreted into the extracellular space. As such, clarified concentrated medium from both A549 WT control and *MMP7* KO cell lines was run on SDS-PAGE, transferred onto nitrocellulose membranes and probed in parallel with ten MMP7 antibodies ( Figure 1). Antibodies were then assessed for their ability to detect intracellular MMP7 using protein lysates from WT control and *MMP7* knockout cells ( Figure 2). MMP7 expression was detected in the protein lysates but required a longer exposure time, indicating that MMP7 is predominantly secreted by A549 cells. Therefore, the performance of antibodies by immunofluorescence and flow cytometry was not evaluated in this study.

**Figure 3.**
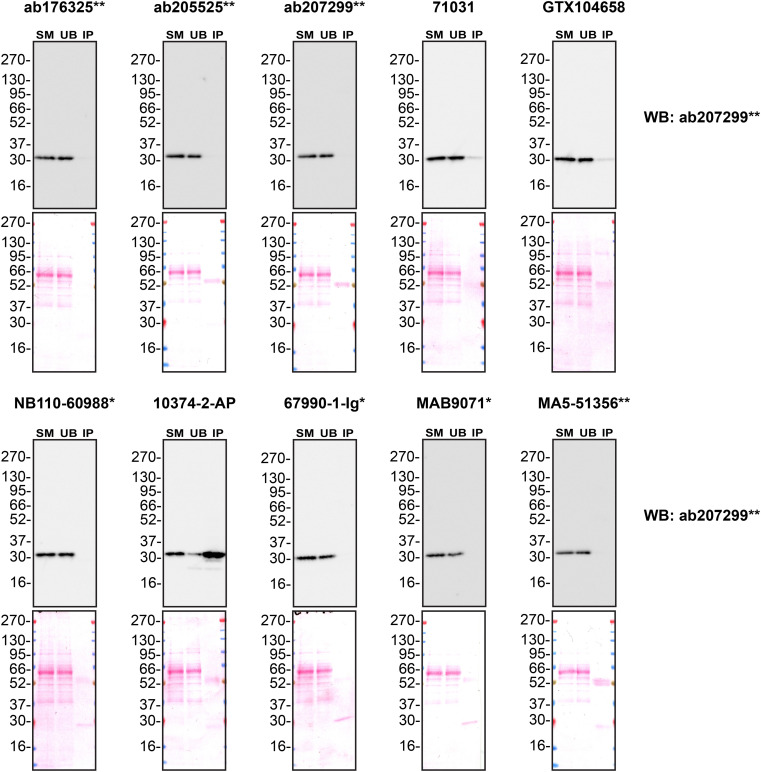
MMP7 antibody screening by immunoprecipitation of culture medium. Conditioned culture medium was collected from A549 WT cells, and immunoprecipitation was performed for 18 hours using 0.5 mg of protein and 2.0 μg of the indicated MMP7 antibodies pre-coupled to Dynabeads protein A or protein G. Samples were washed and processed for western blot with the anti-MMP7 ab207299** diluted at 1/1000. The Ponceau stained transfers of each blot are shown. SM = 4% starting material; UB = 4% unbound fraction; IP = immunoprecipitate. *Monoclonal antibody; **Recombinant antibody.

We next assessed the ability of all ten antibodies to capture MMP7 from A549 culture medium by immunoprecipitation, followed by western blot analysis. For the immunoblot, a specific MMP7 antibody identified previously ( Figure 1) was used. Equal amounts of the starting material (SM) and unbound fraction (UB), along with the complete immunoprecipitated (IP) eluate, were separated by SDS-PAGE ( Figure 3).

In conclusion, we screened ten MMP7 commercial antibodies by western blot and immunoprecipitation by comparing the signal produced using human A549 WT and *MMP7* KO cells. High-quality and renewable antibodies capable of successfully detecting MMP7 were identified.

### Limitations

Inherent limitations are associated with the antibody characterization platform used in this study. Firstly, the YCharOS project focuses on renewable (recombinant and monoclonal) antibodies and does not test all commercially available MMP7 antibodies. YCharOS partners provide approximately 80% of all renewable antibodies, but some top-cited polyclonal antibodies may not be available through these partners. We encourage readers to consult vendor documentation to identify the specific antigen each antibody is raised against, where such information is available.

Secondly, the YCharOS effort employs a non-biased approach that is agnostic to the protein for which antibodies have been characterized. The aim is to provide objective data on antibody performance without preconceived notions about how antibodies should perform or the molecular weight that should be observed in western blot. As the authors are not experts in MMP7, only a brief overview of the protein’s function and its relevance in disease is provided. MMP7 experts are invited to analyse and interpret observed banding patterns in western blots. Thirdly, YCharOS experiments are not performed in replicates primarily due to the use of multiple antibodies targeting various epitopes. Once a specific antibody is identified, it validates the protein expression of the intended target in the selected cell line, confirms the lack of protein expression in the KO cell line and supports conclusions regarding the specificity of the other antibodies. All experiments are performed using master mixes, and meticulous attention is paid to sample preparation and experimental execution. In instances where antibodies yield no signal, a repeat experiment is conducted following titration. Additionally, our independent data is performed subsequently to the antibody manufacturers internal validation process, therefore making our characterization process a repeat.

Lastly, as comprehensive and standardized procedures are respected, any conclusions remain confined to the experimental conditions and cell line used for this study. The use of a single cell type for evaluating antibody performance poses as a limitation, as factors such as target protein abundance significantly impact results. Additionally, the use of cancer cell lines containing gene mutations poses a potential challenge, as these mutations may be within the epitope coding sequence or other regions of the gene responsible for the intended target. Such alterations can impact the binding affinity of antibodies. This represents an inherent limitation of any approach that employs cancer cell lines.

## Methods

The standardized protocols used to carry out this KO cell line-based antibody characterization platform was established and approved by a collaborative group of academics, industry researchers and antibody manufacturers. The detailed materials and step-by-step protocols used to characterize antibodies in western blot, immunoprecipitation and immunofluorescence are openly available on Protocols.io (DOI: dx.doi.org/10.21203/rs.3.pex-2607/v1).

### Antibodies

All MMP7 antibodies are listed in Table 2, together with their corresponding Research Resource Identifiers (RRID), to ensure antibodies are cited properly. ^10^ Secondary antibodies used in this study are provided in Table 3. To ensure consistency with manufacturer recommendations and account for proprietary formulations (where antibody concentrations are not disclosed), antibody usage is reported as dilution ratios rather than absolute concentrations.

**Table 2.** Summary of the MMP7 antibodies tested.

Company	Catalogue number	Lot number	RRID (Antibody registry)	Clonality	Clone ID	Host	Stock concentration (μg/mL)	Vendor recommended applications
Abcam	ab176325 **	1081135-3	AB_3668828	Recombinant Monoclonal	EPR1251(2)	Rabbit	1636	FC-Intra, WB
Abcam	ab205525 **	1029001-2	AB_2861279	Recombinant Monoclonal	EPR17888-101	Rabbit	1537	IHC, WB
Abcam	ab207299 **	1001762-3	AB_2894849	Recombinant Monoclonal	EPR17888-71	Rabbit	1370	ICC/IF, IHC, WB
Cell signaling Technology	71031	1	AB_2799796	Polyclonal	-	Rabbit	160	WB
GeneTex	GTX104658	44377	AB_1241062	Polyclonal	-	Rabbit	450	ELISA, IHC, WB
Novus Biologicals	NB110-60988 *	2020071501	AB_925513	Monoclonal	MM0022-4C21	Mouse	200	IHC, WB
Proteintech	10374-2-AP	00079668	AB_2144452	Polyclonal	-	Rabbit	600	ICC/IF, IHC, WB
Proteintech	67990-1-Ig *	10022681	AB_2918739	Monoclonal	2E6D6	Mouse	1000	ELISA, ICC/IF, IHC, WB
R & D Systems	MAB9071 *	DRG0624021	AB_2282021	Monoclonal	111433	Mouse	500	IHC, IP, WB
Thermo Fisher Scientific	MA5-51356 **	ZJ4521735A	AB_3093027	Recombinant Monoclonal	8T4R8	Rabbit	1000	ELISA, IHC, WB

ELISA = Enzyme-linked immunosorbent assay, FC = Flow cytometry, ICC/IF = Immunocytochemistry/immunofluorescence, IHC = immunohistochemistry, IP = immunoprecipitation, WB = Western blot.

* Monoclonal antibody.

** Recombinant antibody.

**Table 3.** Summary of the secondary antibodies used.

Company	Secondary antibody	Catalogue number	RRID (Antibody registry)	Clonality	Application	Stock concentration (μg/mL)	Working concentration (μg/mL)
Proteintech	HRP-Goat Anti-Rabbit Secondary Antibody (H+L)	RGAR001	AB_3073505	Recombinant Polyclonal	Western blot	1000	0.1
Proteintech	HRP-Goat Anti-Mouse Secondary Antibody (H+L)	RGAM001	AB_3068333	Recombinant Polyclonal	Western blot	1000	0.1
Abcam	Veriblot	ab131366	AB_2892718	Not specified	Immunoprecipitation	40	0.04

### Cell culture

All cell lines used in this study are listed in Table 1, alongside their corresponding RRIDs, to ensure proper citation. ^11^ Cells were cultured in DMEM high-glucose (Capricorn Scientific #DMEM-HPSTA) supplemented with 10% fetal bovine serum (Thermo Fisher Scientific #A5256801) and 1% antibiotic/antimycotic solution (Capricorn Scientific #AAS-B). All cell lines used in this study were routinely tested for mycoplasma contamination and were confirmed to be mycoplasma-free.

### CRISPR/Cas9 genome editing

The A549 *MMP7* KO clone was generated using low-passage cells. Prior to ribonucleoprotein transfection, 20,000 cells were plated in each well of a 48-well plate and incubated overnight to allow attachment. in vitro guide RNA was generated with the HighYield T7 sgRNA synthesis kit (Jena Bioscience #RNT-105), followed by incubation with DNase I-XT (New England Biolabs #M0570) and quick CIP (New England Biolabs #M0525) to remove DNA and 5’ phosphorylation respectively. RNA was purified using the Monarch spin RNA cleanup kit (New England Biolabs #T2040) and 125 ng of guide RNA (target sequence: TCAAAGGCTTTAAACATGTG) was combined with 625 ng of spCas9. Ribonucleoprotein transfection was then performed using the Lipofectamine CRISPRMAX Cas9 transfection reagent (Thermo Fisher Scientific #CMAX000008) according to the manufacturer’s protocol. The culture medium was replaced the following day, and single-cell isolation was performed on day three. Single cells were plated into 96-well plates and clonally expanded.

### Antibody screening by western blot

A549 WT and *MMP7* KO cells were washed three times in Hanks’ balanced salt solution (HBSS) (Capricorn Scientific #HBSS-2A) and serum deprived for 48-hours in DMEM media without phenol red (Thermo Fisher Scientific #21063029) supplemented with 1% antibiotic/antimycotic solution. Culture medium was then collected and centrifuged for 500 × g, for 10 minutes at 4°C to eliminate cells and larger contaminants, then for 4500 × g, for 10 minutes at 4°C to eliminate smaller contaminants. Conditioned medium was then concentrated by centrifugation at 4000 × g for 30 minutes at 4°C using Amicon Ultra 15 mL centrifugal filters with a 3 kDa molecular weight cut off (Sigma Aldrich #UFC900396). Culture media was then supplemented with 1× protease inhibitor cocktail (Cell Signaling Technology #7012).

For lysate preparation, A549 WT and *MMP7* KO cells were washed three times in phosphate buffered saline (PBS) (Thermo Fisher Scientific #70011044) and lysed in RIPA buffer containing 1× of protease inhibitor cocktail, sodium orthovanadate and phenylmethylsulfonyl fluoride (Santa Cruz Biotechnology #sc-24948). Lysates were sonicated (40% amplitude for 5 seconds) three times and incubated for 30 minutes on ice prior to centrifugation at 20,000 × g for 1 hour at 4°C.

Protein concentration was confirmed using the Pierce BCA protein assay (Thermo Fisher Scientific #23225) and 30 μg of protein was used for both protein lysates and concentrated cell culture medium. Samples were combined with Laemmli sample buffer (Bio-Rad #1610747) containing 2-mercaptoethanol (final concentration 355 mM) (Sigma Aldrich #M7522) before being heated at 65°C for 10 minutes. Samples were then loaded in precast 4-20% WedgeWell Tris-Glycine Plus midi gels (Thermo Fisher Scientific #WTG42020BOX) alongside Prime-Step prestained broad range protein ladder (BioLegend #773302). SDS-PAGE was then performed in SureLock Tandem Midi Gel tanks (Thermo Fisher Scientific #STM1001) and run at 200V for 1 hour with Tris/Glycine/SDS buffer (Bio-Rad #1610772). Proteins were then transferred to 0.2μm supported nitrocellulose membranes (Cytiva #10600015) using a Criterion blotter with plate electrodes (Bio-Rad #17004070) run at 85V for 45 minutes. Proteins on the blot were then visualised with Ponceau S staining (Thermo Fisher Scientific #161470250) which was scanned to show alongside individual western blots. Blots were blocked with 5% milk for 1 hour except for antibody 71031 which was blocked in 5% BSA in Tris-buffered saline containing 1% Tween 20 (TBST) (Thermo Fisher Scientific #J77500.K2). Primary antibodies were then incubated overnight at 4°C in 5% milk TBST with gentle shaking. Following three ten-minute washes with TBST, horseradish peroxidase (HRP) conjugated secondary antibodies were incubated at a dilution of 1/10000 (0.1 μg/mL) in TBST with 5% milk for 1 hour at room temperature followed by three ten-minute washes with TBST. Membranes were then incubated with either Pierce ECL (Thermo Fisher Scientific #32106) for 1 minute or Clarity Western ECL substrate (Bio-Rad #1705061) for 5 minutes prior to detection with the ImageQuant LAS 4000.

### Antibody screening by immunoprecipitation

Antibody-bead conjugates were prepared by adding 2 μg of antibody to 1 mL of Pierce IP Lysis Buffer (25 mM Tris-HCl pH 7.4, 150 mM NaCl, 1 mM EDTA, 1% NP-40 and 5% glycerol) (Thermo Fisher Scientific #87788) in a microcentrifuge tube, together with 30 μL of protein A (for rabbit antibodies) or protein G (for mouse antibodies) (Thermo Fisher Scientific #10002D and #10004D respectively). Tubes were rocked for 1 hour at 4°C followed by two washes to remove unbound antibody. Culture media from A549 WT were collected as described in the western section above. 0.5 mL aliquots at 1 mg/mL of culture medium were incubated with an antibody-bead conjugate for 18 hours at 4°C. The unbound fractions were collected, and beads were subsequently washed three times with 1.0 mL of IP buffer and processed for SDS-PAGE and western blot on precast midi 4-20% Tris-Glycine polyacrylamide gels.

## Data Availability

Zenodo: Dataset for the MMP7 antibody screening study contains the underlying data included in a study which characterized ten commercially available antibodies against Matrix metallopeptidase 7 (MMP7) by immunoblot (Western blot) and immunoprecipitation, using a knockout based validation. https://doi.org/10.5281/zenodo.17925527. Data are available under the terms of the Creative Commons Attribution 4.0 International license (CC-BY 4.0).
