# Supplementary material for: A guide to selecting high-performing antibodies for MMP7 (UniProt ID: P09237) for use in western blot and immunoprecipitation
Source: F1000Res. 2026 Jan 19;15:74. [Version 1] doi: 10.12688/f1000research.175973.1 (PMC12949374; doi:10.12688/f1000research.175973.1)
Supplement: Peer review history

# Reviewer report: Reviewer response for version 1

**DOI:** 10.5256/f1000research.193998.r451625

**Published:** 2026-02-27

**Authors:** Christian Tiede

**Affiliations:** 1 University of Leeds, Leeds, England, UK

In this data note, the authors systematically compare the performance and specificity of ten commercially available anti-MMP7 antibodies using Western blot and immunoprecipitation assays. Because intracellular MMP7 levels are low, immunofluorescence staining could not be performed. The study reveals substantial differences among the antibodies in their ability to detect MMP7 in both Western blot and immunoprecipitation applications. This work is particularly valuable because it not only identifies reliable primary anti-MMP7 antibodies but also provides practical guidance for selecting appropriate secondary antibodies for immunoprecipitation. As a result, the findings will benefit not only the MMP7 research community but also researchers working with similar detection methods.

 For completeness, it is recommended that the authors include the dilution information for each primary anti-MMP7 antibody in the Methods section rather than in the captions of Figures 1 and 2.

Are sufficient details of methods and materials provided to allow replication by others?

Yes

Is the rationale for creating the dataset(s) clearly described?

Yes

Are the datasets clearly presented in a useable and accessible format?

Yes

Are the protocols appropriate and is the work technically sound?

Yes

Reviewer Expertise:

phage display, protein engineering, antibody alternative scaffolds.

I confirm that I have read this submission and believe that I have an appropriate level of expertise to confirm that it is of an acceptable scientific standard.

# Reviewer report: Reviewer response for version 1

**DOI:** 10.5256/f1000research.193998.r451624

**Published:** 2026-01-30

**Authors:** Jieya Shao

**Affiliations:** 1 Washington University in St Louis, St. Louis, USA

In this article the authors systematically compared the performance and specificity of ten commercially available anti-MMP7 antibodies using two standard experimentais approaches (Western blot and immunoprecipitation). The authors could not perform immunofluorescence staining due to the fact that the target MMP7 is a secreted protein and its intracellular level is too low in most cell lines. This study is part of a large collaborative initiative to characterize commercial antibodies for different human proteins and offer unbiased guidance to researchers who can select the best antibodies for their specific experimental objectives based on their own interpretations. As a Data Note, this paper successfully achieved its goal with rigorously collected and clearly presented high-quality data, and the results are useful for the research community.

 The authors are encouraged to capitalize “Western blot” in the text. Also, in the Methods section, under CRISPR/Cas9 genome editing, the first word (In vitro) should be capitalized.

Are sufficient details of methods and materials provided to allow replication by others?

Yes

Is the rationale for creating the dataset(s) clearly described?

Yes

Are the datasets clearly presented in a useable and accessible format?

Yes

Are the protocols appropriate and is the work technically sound?

Yes

Reviewer Expertise:

Genome integrity, DNA replication stress, cancer biology

I confirm that I have read this submission and believe that I have an appropriate level of expertise to confirm that it is of an acceptable scientific standard.
